# A Case–Control Analysis of Exposure to Traffic and Acute Myocardial Infarction

**DOI:** 10.1289/ehp.9587

**Published:** 2006-10-11

**Authors:** Cathryn Tonne, Steve Melly, Murray Mittleman, Brent Coull, Robert Goldberg, Joel Schwartz

**Affiliations:** 1 Department of Environmental Health and; 2 Department of Epidemiology, Harvard School of Public Health, Boston, Massachusetts, USA; 3 Beth Israel Deaconess Medical Center, Boston, Massachusetts, USA; 4 Department of Biostatistics, Harvard School of Public Health, Boston, Massachusetts, USA; 5 University of Massachusetts Medical School, Worcester, Massachusetts, USA

**Keywords:** air pollution, case–control studies, myocardial infarction, vehicle emissions

## Abstract

**Background:**

Long-term exposure to particulate air pollution has been associated with an increased risk of dying from cardiopulmonary and ischemic heart disease, yet few studies have evaluated cardiovascular end points other than mortality. We investigated the relationship between long-term exposure to traffic and occurrence of acute myocardial infarction (AMI) in a case–control study.

**Methods:**

A total of 5,049 confirmed cases of AMI were identified between 1995 and 2003 as part of the Worcester Heart Attack Study, a community-wide study examining changes over time in the incidence of AMI among greater Worcester, Massachusetts, residents. Population controls were selected from Massachusetts resident lists. We used cumulative traffic within 100 m of subjects’ residence and distance from major roadway as proxies for exposure to traffic-related air pollution. We estimated the relationship between exposure to traffic and occurrence of AMI using logistic regression, and we adjusted for the following potential confounders: age, sex, section of the study area, point sources emissions of particulate matter with aerodynamic diameter < 2.5 μm, area socioeconomic characteristics, and percentage of open space.

**Results:**

An increase in cumulative traffic near the home was associated with a 4% increase in the odds of AMI per interquartile range [95% confidence interval (CI), 2–7%], whereas living near a major roadway was associated with a 5% increase in the odds of AMI per kilometer (95% CI, 3–6%).

**Conclusions:**

hese results provide support for an association between long-term exposure to traffic and the risk of AMI.

Cohort studies in the United States and Europe have shown associations between long-term exposure to particulate air pollution and increased mortality, particularly for cardiopulmonary and ischemic heart disease–related deaths (Dockery et al. 1993; Hoek et al. 2002; Pope et al. 2002, 2004). Several of these cohort studies were limited, however, by the number of deaths from a particular cause, limiting their ability to identify particular diseases at higher risk from exposure to air pollution. Case–control studies, in contrast, allow the accumulation of a large number of cases of a specific disease. Moreover, the coding of specific causes of death on death certificates has been shown to have relatively low specificity, (Coady et al. 2001; Pope et al. 2004), further limiting the interpretation of cohort studies that have examined the association between air pollution and specific causes of death. Furthermore, relatively few studies have evaluated the effects of long-term exposure on cardiovascular end points other than mortality, such as acute myocardial infarction (AMI). The evidence from the few case–control studies evaluating long-term exposure to traffic-related air pollution and myocardial infarction has been inconclusive (Grazuleviciene et al. 2004; Rosenlund et al. 2006).

Most cohort studies to date that assessed the association between air pollution and risk of disease or mortality have also had limited ability to account for geographic variation in exposure within cities (e.g., Dockery et al. 1993; Pope et al. 2002). Recent studies have suggested that this variation is quite important in assessing the effects of long-term exposure to air pollution. Epidemiologic studies that have accounted for intraurban variability in exposure due to local sources, such as traffic, have generally found larger effect estimates than studies that relied on between-city comparisons (Hoek et al. 2002; Jerrett et al. 2005). Although there is relatively little spatial variation in particulate matter < 2.5 μm aerodynamic diameter (PM_2.5_) concentrations within an urban area (Burton et al. 1996), finer spatial variability has been shown for components of PM_2.5_ emitted primarily by motor vehicles (Lena et al. 2002). Such findings indicate that the health effects of traffic pollutants can be partially separated from those of background pollution levels by focusing on the intraurban spatial variability in components of PM_2.5_. Moreover, a recent case–crossover study found that exposure to motor vehicle traffic was associated with a nearly 3-fold increase in the odds of AMI within 1 hr of exposure, suggesting that traffic particles may be an important etiologic factor associated with the occurrence of AMI (Peters et al. 2004). However, it is unclear whether long-term as well as short-term exposure to traffic pollutants is associated with the development of AMI.

We focused on a single metropolitan area, in which PM_2.5_ is relatively spatially homogeneous (Burton et al. 1996), and used a case–control study design to evaluate the association between indicators of traffic and AMI. We hypothesized that local traffic near the home and residence near a major roadway—both proxies for long-term exposure to traffic pollutants—increase the odds of AMI.

## Materials and Methods

### Selection of cases and controls

The present investigation was limited to residents of the greater Worcester, Massachusetts, metropolitan area who were ≥25 years of age. Worcester is a moderate-size city in central Massachusetts, with a metropolitan area population of approximately 478,000 according to the 2000 census. Independently validated cases were identified as part of the Worcester Heart Attack Study, an ongoing community-wide investigation examining changes over time in the incidence, hospital, and long-term case-fatality rates of Worcester-area residents hospitalized with confirmed AMI (Goldberg et al. 1986, 1999). Possible cases of AMI were admitted to any of 11 acute care general hospitals in the Worcester metropolitan area during the study years 1995, 1997, 1999, 2001, and 2003. Medical records of all patients with a discharge diagnosis of AMI (*International Classification of Diseases, Ninth Revision* code 410) (National Center for Health Statistics 2005) were reviewed and independently validated according to preestablished diagnostic criteria described previously (Goldberg et al. 1986; Goldberg et al. 1999). Briefly, these criteria included a suggestive clinical history, increased cardiac enzyme levels above each hospital’s normal range, and serial electrocardiographic findings indicative of AMI. To be included in the study, at least two of these criteria had to be met. Information was abstracted by trained physicians and nurses from hospital medical records about patients’ demographic characteristics, medical history, and clinical characteristics.

Population controls were randomly selected from resident lists published in 2003. Resident lists are published annually by each town in Massachusetts and include all residents ≥17 years of age (Commonwealth of Massachusetts 2005). Inclusion in the list is mandated by state law and is based on response to a mailing or visit by the town registrar. Information included in the lists varied from town to town, but at a minimum included name, street address, sex, and year of birth. Twice as many controls were selected for the present investigation as there were cases. Controls were frequency matched to cases on the basis of age (in 10-year categories), sex, and section of the study area (one of three regions of roughly equal population), such that controls were selected independently of residential location within section. The sections were central Worcester, the northern suburbs, and the southern suburbs.

Cases’ residential addresses at the time of AMI were collected from the review of hospital medical records, and the controls’ residential addresses were extracted from the resident lists. Addresses were sent to a commercial firm for geocoding (Mapping Analytics, Rochester, NY). Study subjects who could not be geocoded accurately at the block group level were excluded (*n* = 37). The study was approved by the Committee for the Protection of Human Subjects at the University of Massachusetts Medical School and the Human Subjects Committee at the Harvard School of Public Health.

### Assessment of exposure and covariates

We considered two different measures of exposure to traffic: distance to nearest major roadway (limited-access highway or multilane highway) according to the Mass Highway road database [Executive Office of Transportation and Construction (EOT) 2003], and cumulative traffic at subjects’ residence using ArcGIS software (version 9; ESRI, Redlands, WA). To represent local scale traffic, we estimated cumulative traffic by creating a 100-m radius buffer around each subject’s residence and summing the product of road segment length and estimated annual average daily traffic from the Mass Highway 2002 road inventory falling within the buffer (EOT 2003). We chose a buffer of 100-m radius based on previous findings which indicated that a 100-m buffer was the most predictive of PM_2.5_ absorbance (a surrogate of traffic particles) and nitrogen dioxide, also a traffic pollutant (Hochadel et al. 2006). Although we *a priori* chose a buffer radius of 100 m based on the published literature, we also evaluated the associations of interest for a buffer size of 200 and 300 m as a sensitivity analysis. Measures of annual average daily traffic are based on actual traffic counts for major roadways but are only estimated according to regional traffic for more local roads.

Data on 1999 PM_2.5_ emissions from point sources were downloaded from the U.S. Environmental Protection Agency (EPA) National Emissions Inventory (NEI) database (U.S. EPA 1999). Approximately 90 point sources of PM_2.5_ lay within the study area, including but not limited to industrial facilities, hospitals and medical centers, and education institutions. We estimated the PM_2.5_ emissions per unit area using a kernel function to fit a smoothly tapered surface to each NEI point source using the kernel density tool in ArcGIS (Silverman 1986). With this approach, the emissions value is highest at the location of the source and diminishes with increasing distance until 10 km from the source, where the emissions value is set to zero. We divided the study area into 50 m^2^ raster cells and calculated the estimated emission density for each cell by summing the surfaces from all sources where they overlay the cell center. The estimated emission density was extracted at the location of each subject’s residence.

Because exposure to particles varies spatially, we examined several spatially varying potential confounders. We downloaded data on open space from the MassGIS website (MassGIS 2005). We considered the following open space variables: percentage of census tract that is conservation or recreation area and distance (in meters) to nearest recreation or conservation area. We defined population density as the total block group population in the 2000 census per area of dry land in square kilometers, based on MassGIS data (MassGIS 2003). Moreover, we matched cases and controls by area of residence to further control for confounding by differences other than traffic between the downtown city of Worcester and its outlying regions.

We derived area-based socioeconomic measures from the 2000 U.S. Census at the block group level (U.S. Census Bureau 2002). Census block groups are designed to have an optimal size of 1,500 people, and have an area smaller and, by design, more homogeneous socioeconomically than a census tract (U.S. Census Bureau 2005). We used the following census-derived measures of socioeconomic position (SEP): median income (median household income in 1999); percentage of persons below the federally defined poverty line; and percentage of persons ≥25 years of age whose highest degree was less than a high school diploma or its equivalent.

### Statistical analysis

First, we created logistic regression models using the SAS 9.1 software package (SAS Institute Inc., Cary, NC) to estimate the odds ratio (OR) for each spatial variable while adjusting only for the matching factors (age, sex, and section of study area). Although we matched on age in 10-year groups, we adjusted for age more finely by including it as a continuous, linear term in the regression models. We initially considered several different measures of SEP and open space in the models adjusting only for the matching factors; however, in the fully adjusted model we included only the single strongest predictor of AMI from among the open space and SEP variables. We then evaluated the sensitivity of the traffic effect estimates to adjustment by alternative SEP and open space variables. Measures of cumulative traffic within the 100-m radius of the home, open space, and population density were highly skewed and were log-transformed in all regression models. We confirmed the adequacy of a linear term to model age, distance from major roadway, and cumulative traffic using penalized spline regression in the R software package (Free Software Foundation, Boston, MA). From a set of potential confounders of the association between traffic exposure and AMI that were selected *a priori*, we included variables if they were predictors of AMI in the model adjusting for only the matching factors and were correlated with exposure to traffic among the controls.

In the fully adjusted model, we included both cumulative traffic, a measure of local traffic, and distance to major roadway, representing traffic emissions on a larger spatial scale. We accounted for remaining spatial autocorrelation resulting from inadequately modeled or unmeasured spatially distributed risk factors by including a random intercept for each block group and specifying a spatial covariance structure in which block groups whose centroids are farther apart are less correlated. This model was fit using the GLIMMIX procedure in SAS (SAS Institute Inc.). We also repeated the analysis to estimate the association between exposure to traffic and occurrence of initial AMI by restricting to cases who experienced their first AMI event. This analysis was performed because individuals who experienced a recurrent coronary event are at greater risk of AMI than those who experienced their first event.

## Results

Table 1 presents the distribution of selected covariates and exposure to traffic for cases and controls. In the regression models that were adjusted for only the matching factors, living near a major roadway and a higher level of cumulative traffic were significantly associated with occurrence of AMI (Table 2). PM_2.5_ point source emissions, percentage of block group residents living below the poverty line, percentage with less than a high school education, as well as population density were also significantly and positively associated with occurrence of AMI. Household median income of block group, distance from nearest recreation area, and percentage of census tract that was recreation or conservation space were significantly and negatively associated with AMI.

In the fully adjusted model, we included the following potential confounders of the association between exposure to traffic and AMI in addition to the matching factors: percentage of census tract that was conservation area, percentage of block group residents living below the poverty line, population density, and PM_2.5_ point source emissions. The associations between cumulative traffic and living near a major roadway were attenuated after adjustment for confounding but remained significant (Table 2). In the fully adjusted model, the odds of AMI significantly decreased with increasing open space, and significantly increased with increasing percentage of block group residents living below the poverty line. Population density and PM_2.5_ emissions density from point sources were no longer predictive of AMI.

### Sensitivity analysis

The effect estimates for living near a major roadway and cumulative traffic near the home were not sensitive to exclusion of the eight controls who were also cases, inclusion of higher-order interactions between the matching factors, or adjustment for confounding using alternative SEP or open space measures. Nonlinear terms for age, distance from major roadway, and cumulative traffic did not significantly improve the model fit. Further adjustment for remaining spatial autocorrelation changed the effect estimates of both exposures by < 2%. Cumulative traffic within a 100-m buffer radius was more strongly associated with AMI than was cumulative traffic using the 200-and 300-m buffer sizes.

### Subgroup analysis and effect modification

The effect estimates for both measures of exposure to traffic were similar when only cases of first AMI were included. There was no evidence that the effect estimates for cumulative traffic near the home or distance from roadway differed by sex or quintile of percentage of block group residents living below the poverty line. However, we did find that the effect estimate for cumulative traffic near the home was larger for younger individuals: A higher level of cumulative traffic increased the odds of AMI by 9.7% per interquartile range (IQR) for individuals < 65 years of age [95% confidence interval (CI), 6.5–12.9] whereas the increase in odds among individuals 65–74 years of age was 4.5% (95% CI, 1.7–7.4). There was no association between cumulative traffic and AMI among individuals ≥75 years of age (OR = 1.0; 95% CI, 0.98–1.0). Similar associations according to age were observed among cases with an initial AMI.

## Discussion

In this population-based study, we observed a significant increase in the odds of AMI associated with increasing exposure to traffic within 100 m of subjects’ residence and with living near major roadways. An association between transient exposure to traffic and the onset of AMI within the preceding hour has been previously observed (Peters et al. 2004). Our findings suggest that long-term exposure to elevated levels of traffic may also increase the occurrence of AMI, although these observations must be considered in light of the study limitations. Moreover, the present analysis indicates there is sufficient intraurban variation in long-term exposure to traffic in the greater Worcester area to detect a differential risk of AMI.

A growing body of evidence supports the use of cumulative traffic near the home and distance from major roadways as proxies for exposure to traffic-related air pollution. Distance from motorways as well as traffic density have been shown to be significant predictors of outdoor measurements of PM absorbance and NO_2_ (Brauer et al. 2003; Hochadel et al. 2006; Hoek et al. 2001; Janssen et al. 2001). Furthermore, previous investigations have shown that concentrations of traffic-related air pollutants drop off to the local background concentration between 100 and 150 m from the roadside (Roorda-Knape et al. 1998), indicating that a 100-m buffer is a reasonable size to capture local traffic-related air pollution. A recent study by Hochadel and colleagues (2006) provides additional support for our choice of buffer size. After evaluating a wide range of buffer sizes, these investigators found cumulative traffic within a 100-m radius buffer was the most predictive of both measured PM_2.5_ absorbance and NO_2_. The model that best predicted measured PM_2.5_ absorbance included both cumulative traffic within a 100-m buffer and distance to the nearest highway, indicating that the two proxies we selected in the present investigation effectively represent traffic-related air pollution at different spatial scales.

That the observed association can be attributed to traffic-related air pollution also appears plausible in light of previous investigations, particularly those that evaluated the association between long-term exposure to traffic and mortality as well as the association between short-term increases in air pollution and development of AMI. Hoek and colleagues (2002) found that living within 100 m of a freeway or within 50 m of a major urban road in the Netherlands was associated with dying from cardiopulmonary disease, which includes AMI [relative risk (RR) = 1.95; 95% CI, 1.09–3.51]. Within the same model, a 10-μg/m^3^ increase in black smoke (representing the urban background) was associated with a RR of 1.34 (95% CI, 0.68–2.64). Living within 100 m of a freeway or within 50 m of a major urban road was also associated with increased all-cause mortality among residents of Ontario (RR = 1.18; 95% CI, 1.02–1.38) (Finkelstein et al. 2004). Time-series studies have found significant associations between traffic-related air pollutants (black smoke, NO_2_, carbon monoxide) and daily hospital admissions for AMI (Lin et al. 2003; Poloniecki et al. 1997). Similarly, a multicity case–crossover analysis found increasing concentrations of PM with aerodynamic diameter < 10 μg/m^3^ were positively associated with hospital admissions for AMI (Zanobetti and Schwartz 2005). In addition, developing literature on the mechanistic basis for an association between traffic particles and increased risk of either developing or dying from AMI supports the toxicologic plausibility of our findings (Gurgueira et al. 2002; Künzli et al. 2005; Lippmann et al. 2005; Sun et al. 2005; Suwa et al. 2002). In contrast, no association was observed between long-term average air pollution exposure modeled from historical emission data and overall incidence of myocardial infarction in a recent case–control study (Rosenlund et al. 2006). However, the investigators observed a stronger association between ever living near a traffic hot spot and occurrence of AMI, suggesting that exposure assessment approaches that specifically account for the influence of local traffic patterns may characterize important variation in exposure not captured by dispersion models based on historical emission data.

Alternatively, the observed association between long-term exposure to traffic and increased odds of AMI may be caused partly by attributes of the residential environment that may affect the development of AMI other than traffic-related air pollution. A meta-analysis of three studies evaluating the association between traffic noise and AMI observed an elevated but nonsignificant association (RR = 1.03) between the two (van Kempen et al. 2002). A similar study observed a positive, nonsignificant association between chronic exposure to traffic noise and AMI (Babisch et al. 2005); however, none of the studies adjusted for exposure to traffic-related air pollution. In addition to noise, other attributes of the residential environment that may influence the risk of AMI include availability and relative cost of healthy foods and prevalence of advertising for tobacco, which influence the spatial distribution of diet and smoking habits (Diez Roux 2003; Luke et al. 2000; Morland et al. 2002). However, such factors may represent specific pathways by which neighborhood socioeconomic characteristics influence risk of AMI, rather than confounders of the association between exposure to traffic-related air pollution and AMI.

The socioeconomic characteristics of both individuals and the neighborhoods they live in have been shown to be important predictors of cardiovascular health (Diez-Roux et al. 2001; Smith et al. 1998; Stjarne et al. 2002). We adjusted for only socioeconomic characteristics at the block group level. Although block groups are designed to be relatively homogeneous with respect to socioeconomic characteristics, variation in the socioeconomic circumstances of individuals within the block group remains. Individuals living within hundreds of meters of high-traffic roads may have lower SEP than the average SEP of their block group. Higher exposure to traffic-related air pollution may, in fact, be one of the pathways by which individuals with lower SEP are at elevated risk for AMI. However, because we did not have data on individuals’ SEP, we were unable to separate the association of interest from other pathways by which individuals’ SEP increases the risk of AMI that are not mediated by exposure to traffic-related air pollution.

Individuals’ established and novel risk factors for cardiovascular disease may also be correlated with place of residence within a neighborhood, and also could explain, in part, our observed association between exposure to traffic and increased risk of AMI. To explore the degree to which smoking was associated with exposure to traffic, we had previously geocoded addresses of 804 subjects in the Myocardial Infarction Onset study (Mittleman et al. 1993). Although these subjects were primarily residents of the greater Boston, Massachusetts, area rather than from the greater Worcester area, distance from roadway was not predictive of smoking status.

We observed a stronger association between exposure to traffic and occurrence of AMI among younger individuals. The observed effect modification by age could be caused by differences by age in the extent of exposure misclassification or residual confounding rather than biologic differences in susceptibility to traffic. Processes underlying the observed effect modification require further investigation.

We conceptualized open space as a potential confounder in this analysis because it may partially represent individuals’ level of physical activity. Access to parks, walking and jogging trails, and enjoyable scenery have been associated with physical activity behavior (Brownson et al. 2001). However, the percentage of open space near the home may also represent aspects of exposure to traffic or point-source emissions not captured by the GIS-based measures we used. We therefore presented the associations between the GIS-derived measures of exposure to traffic and AMI with and without adjustment for open space in Table 2.

### Study limitations

We used cumulative traffic near the home and distance from roadway as proxies for long-term personal exposure to traffic-related air pollution because data on individuals’ long-term exposures were not available. Therefore, misclassification of exposure is an important limitation of the present analysis. Personal exposure to traffic-related air pollution is influenced by time–activity patterns, air conditioning use, and time spent in locations other than at home, such as the workplace. These aspects of personal exposure are not reflected by a surrogate representing pollution outdoors at the residential location. For example, residents who live near major roadways may be less likely to open their windows, have access to air conditioning due to lower socioeconomic position, and may have shorter commute times compared with individuals who live far from major roadways. A further limitation of traffic indicators is that they do not account for wind direction or topography, and therefore assume that dispersion of pollutants from roadways is the same in all directions. However, it is likely that most misclassification of personal exposure introduced by relying on traffic indicators is nondifferential with respect to AMI and will lead to an underestimate of the association. Because we did not have data on previous residential history, we were unable to take into account how long people lived at their current residence, which also could have produced misclassification of long-term exposure. This loss of precision in exposure could be greater for controls because controls were selected from the 2003 resident lists, and exposure was assigned to controls based on their residential address at that time; this may result in a potential bias of the association between traffic-related air pollution and AMI.

Cases of AMI were identified through a systematic surveillance approach that collects data on greater Worcester residents who sought care for AMI at hospitals in the greater Worcester area and beyond. Therefore, data were not available for patients with silent or unrecognized AMI, which can account for more than a quarter of all AMIs (Kannel and Abbott 1984), or on out-of-hospital sudden cardiac deaths. However, it is unclear whether the care-seeking patterns for these patients would depend on where an individual lives.

In conclusion, we observed a significant association between exposure to traffic near the place of residence and the occurrence of AMI. We used surrogates of long-term exposure to traffic-related air pollution in the present analysis; this potential association requires further investigation using more direct measures of individual long-term exposure to traffic pollutants. Moreover, the influence of traffic on AMI could not be separated from that of other aspects of the residential environment in which individuals at risk for AMI live. Confirmation of this potential association in populations for which additional individual-level information on SEP and cardiovascular risk factors is available would be particularly relevant.

## Figures and Tables

**Table 1:** Characteristics of cases of AMI and population controls.

Characteristic	Cases (*n* = 5,049)	Controls (*n* = 10,277)
Demographic characteristics		
Age [years (mean ± SD)]	71 ± 14	70 ± 14
Percent male	56.4	56.8
Previous AMI (%)	35.4	—
Open space		
Census tract area that is conservation (%)	5.5	6.2
Distance to conservation area (m)	978	939
Population density (individuals per km^2^)	1,854	1,696
Point-source PM_2.5_ emission density (tons per m^2^ ± SD)	0.31 ± 0.2	0.30 ± 0.2
Block group socioeconomic position		
Median income (US$)	47,595	49,771
Residents living below the poverty line (%)	10.7	9.1
Residents with less than high school education (%)	18	16
Exposure to traffic		
Distance to major roadway (km)		
5th percentile	0.07	0.09
25th percentile	0.43	0.47
Median	0.92	1.06
Mean ± SD	1.6 ± 2.1	1.9 ± 2.5
75th percentile	2.0	2.3
95th percentile	5.7	6.2
Traffic within 100 m of residence (vehicle-km)		
5th percentile	20	19
25th percentile	177	160
Median	369	324
Mean ± SD	1,145 ± 2,130	1,060 ± 2,048
75th percentile	1,080	954
95th percentile	4,639	4,403

**Table 2:** Relative odds of AMI associated with long-term exposure to traffic.

Model	OR (95% CI)
All AMI (*n* = 15,326)	
Adjusted for matching factors	
Cumulative traffic*a* (IQR increase)	1.07 (1.04–1.10)
Living near major roadway (per km)	1.06 (1.05–1.08)
Fully adjusted^b^	
Cumulative traffic^a^ (IQR increase)	1.04 (1.02–1.07)
Living near major roadway	1.05 (1.03–1.06)
Adjusted for spatial autocorrelation^b^,^c^	
Cumulative traffic^a^ (IQR increase)	1.06 (1.03–1.09)
Living near major roadway	1.06 (1.02–1.10)
Initial AMI (*n* = 13,538)	
Adjusted for matching factors	
Cumulative traffic^a^ (IQR increase)	1.07 (1.04–1.11)
Living near major roadway	1.05 (1.04–1.08)
Fully adjusted^b^	
Cumulative traffic^a^ (IQR increase)	1.05 (1.02–1.08)
Living near major roadway	1.04 (1.02–1.06)
Adjusted for spatial autocorrelation^b^,^c^	
Cumulative traffic^a^ (IQR increase)	1.06 (1.03–1.10)
Living near major roadway	1.06 (1.02–1.10)

a Cumulative traffic was modeled as the natural log of the average daily traffic within a 100-m radius of the residence.

b Adjusted for age, section of study area, sex, percent of census tract that was conservation area, percent of block group residents living below the poverty line, and PM_2.5_ point-source emissions density.

c Correlation in the residuals of the regression model resulting from similarities in individuals or areas with respect to unmeasured covariates.
